# Supplementary material for: Effect of Chronic Tibolone Administration on Memory and Choline Acetyltransferase and Tryptophan Hydroxylase Content in Aging Mice
Source: Brain Sci. 2024 Sep 6;14(9):903. doi: 10.3390/brainsci14090903 (PMC11430777; doi:10.3390/brainsci14090903)
Supplement: Supplementary file 1 [file brainsci-14-00903-s001.zip › Table S1. individual data young aged.pdf]

Table S1. Individual recognition index data

Object recognition

Mice number	Young 1 h	Aged 1 h	Young 24 h	Aged 24 h
1	0.768	0.533	0.690	0.514
2	0.665	0.525	0.702	0.509
3	0.662	0.653	0.895	0.516
4	0.670	0.550	0.709	0.528
5	----	---	---	---
6	0.670	0.571	0.703	0.511
7	0.541	0.554	0.705	0.524
8	---	---	---	---
9	0.781	0.552	0.691	0.505

Object recognition in context

Mice number	Young 1 h	Aged 1 h	Young 24 h	Aged 24 h
1	0.722	0.572	0.953	0.553
2	0.752	0.580	0.737	0.562
3	0.743	0.570	0.742	0.564
4	0.742	0.570	0.735	0.570
5	---	---	---	---
6	0.653	0.584	0.868	0.566
7	0.731	0.578	0.737	0.455
8	---	---	---	---
9	0.722	0.579	0.669	0.557

Mice number 5 and 8 died before the test was performed
