# Supplementary material for: Effect of Chronic Tibolone Administration on Memory and Choline Acetyltransferase and Tryptophan Hydroxylase Content in Aging Mice
Source: Brain Sci. 2024 Sep 6;14(9):903. doi: 10.3390/brainsci14090903 (PMC11430777; doi:10.3390/brainsci14090903)
Supplement: Supplementary file 1 [file brainsci-14-00903-s001.zip › Table S2. exploration time Castillo Mendieta 2024.pdf]

Table S2. Individual exploration time (seconds)

Object recognition

Observer 1

Mice number	Young 1 h FO	Young 1 h NO	Aged 1 h FO	Aged 1 h NO	Young 24 h FO	Young 24 h NO	Aged 24 h FO	Aged 24 h NO
**1**	10	10	1	2	2.6	2	1	1.6
**2**	3.4	18	2.2	3.3	4	11.4	2.7	2.7
**3**	7.3	13.1	1.8	1.7	4.5	9.5	2.4	2.3
**4**	4.5	8.1	4.7	4.8	3.7	6.7	2	4
**5**	----	---	---	7.8	---	---	---	---
**6**	11.6	12.3	1.3	2.7	1.1	3.4	1	1.4
**7**	6	1.4	3.4	6	4.4	5.5	4	4.3
**8**	---	---	---	2.6	---	---	---	---
**9**	1.1	8.2	1	2.4	1.2	2.7	4.5	4.8

FO: Familiar Object. NO: New Object

Observer 2

Mice number	Young 1 h FO	Young 1 h NO	Aged 1 h FO	Aged 1 h NO	Young 24 h FO	Young 24 h NO	Aged 24 h FO	Aged 24 h NO
**1**	9	8.5	2	2.5	3	2.5	1.5	1.5
**2**	2.8	15	3.5	4.5	3.5	10.5	3	3
**3**	6.9	12.5	2.5	2	5	10	3	2.5
**4**	5.5	9	4	5.5	4	7.5	2.5	5
**5**	----	---	---	7.5	---	---	---	---

**6**	9.5	11.5	1.3	3	1.5	4	2	1.5
**7**	6.5	3.5	3.4	5.5	5	6	3.5	5
**8**	---	---	---	3	---	---	---	---
**9**	1.5	7.5	1.5	2	2	3	5	5

FO: Familiar Object. NO: New Object

Object recognition in context

Observer 1

Mice number	Young 1 h FO	Young 1 h NO	Aged 1 h FO	Aged 1 h NO	Young 24 h FO	Young 24 h NO	Aged 24 h FO	Aged 24 h NO
**1**	18.1	22.6	1.8	1.5	4	3	0.7	0.5
**2**	5	9.2	1.9	2	2.4	11.3	3.1	3.4
**3**	5.4	18	1.3	2	7.6	12.2	2.5	2.7
**4**	6.7	10.2	6.2	7.6	5.4	6.4	9	8.8
**5**	---	---	---	---	---	---	---	---
**6**	1	1.6	4.1	5.5	1	4.8	3.4	4.2
**7**	7	3.9	3.4	3	2.7	5.5	4.5	6
**8**	---	---	---	---	---	---	---	---
**9**	5.9	8.6	4	7.5	2.1	4.3	2.4	3.6

FO: Familiar Object. NO: New Object

Mice number 5 and 8 died before the test was performed

Observer 2

Mice number	Young 1 h FO	Young 1 h NO	Aged 1 h FO	Aged 1 h NO	Young 24 h FO	Young 24 h NO	Aged 24 h FO	Aged 24 h NO
**1**	15	20	2	2	5	2.5	1	1
**2**	6	10	2	2	2	10.5	3.5	4
**3**	6	17	1.5	2.5	8	11	3	3
**4**	7	9.5	6.5	8	5	7	10	7.5
**5**	---	---	---	---	---	---	---	---
**6**	1.5	2	5	6	1.5	5.5	3	5
**7**	6.5	3.5	4	3.5	3	6	5	5.5
**8**	---	---	---	---	---	---	---	---
**9**	6	9	4	8	2.5	5	3	4

FO: Familiar Object. NO: New Object

Mice number 5 and 8 died before the test was performed
